# New Contraception Update — Annovera, Phexxi, Slynd, and Twirla

**DOI:** 10.1007/s13669-021-00321-4

**Published:** 2022-01-06

**Authors:** Courtney C. Baker, Melissa J. Chen

**Affiliations:** 1 Department of Obstetrics and Gynecology, University of California, Davis, 4860 Y Street, Suite 2500, Sacramento, CA 95817, USA

**Keywords:** Contraception, Progestin-only contraceptives, Vaginal ring, Contraceptive patch, Contraceptive gel

## Abstract

**Purpose of Review:**

In this review, we discuss the efficacy, safety, and benefits of four new contraceptive products available in the USA, specifically Annovera, Phexxi, Slynd, and Twirla.

**Recent Findings:**

Annovera is a vaginal ring releasing ethinyl estradiol and segesterone acetate that can be used for up to one year (13 cycles), offering patients an effective, user-controlled option that may improve contraceptive access for those in low-resource settings or those with barriers to retrieving monthly prescriptions; however, given limited efficacy and safety data in people with body mass index (BMI) > 29 kg/m^2^, clinicians may consider whether Annovera is an appropriate contraceptive method for obese patients if there are other acceptable alternatives. Phexxi prescription-only vaginal gel is a user-controlled, non-hormonal, on-demand contraceptive method that represents a novel addition to the market with its additional uses as a personal lubricant and as a potential microbicide for urogenital infection prevention. Slynd, a drospirenone-only pill, provides more flexibility for delayed or missed pills while maintaining efficacy and a more favorable bleeding profile compared with previously available progestin-only pills. Lastly, Twirla is a transdermal patch releasing ethinyl estradiol and levonorgestrel that offers users an additional option for a user-controlled, combined hormonal contraceptive method without daily dosing; however, prescription is limited to patients with BMI < 30 kg/m^2^ due to decreased efficacy and VTE events in people with obesity.

**Summary:**

The addition of these products expands the available options for pregnancy prevention to address unmet contraceptive needs.

## Introduction

New contraceptive methods increase options for individuals seeking to prevent pregnancy. Contraceptive technology research can address concerns about risks, safety, or convenience of existing methods by developing new routes of administration, utilizing novel steroid hormones or different combinations of steroid hormones, and combining multiple needs into a single product (e.g., pregnancy and infection prevention). Recent additions to the US contraceptive market include a vaginal ring releasing ethinyl estradiol (EE) and segesterone acetate (SA) that can be used cyclically for up to one year, an acid-buffering vaginal gel, a drospirenone-only pill with a wider window for dosing delays, and a transdermal patch releasing EE and levonorgestrel (LNG). In this review, we discuss the efficacy, side effects, risks, benefits, and the potential role of these new products among currently available options to assist clinicians with up-to-date contraception counseling.

## Annovera: the 1-Year Ethinyl Estradiol and Segesterone Acetate Contraceptive Vaginal System

There are several advantages to contraceptive vaginal rings, including non-daily administration, user control, and stable hormone levels compared with daily oral dosing [1]. Most providers are familiar with Nuvaring® (Organon & Co, Jersey City, NJ), a contraceptive vaginal ring containing EE and etonogestrel that is used over one cycle and requires replacement with a new ring each month [2]. Annovera® (Therapeutics MD, Inc, Boca Raton, FL) is a contraceptive vaginal system (CVS) containing EE and SA that was approved by the Food and Drug Administration (FDA) in 2018 [3]. This ring is the first approved contraceptive product to incorporate SA. This progestin has a low oral bioavailability but requires only small doses to achieve pro-gestational activity in sustained-release formulations [4]. In contrast to Nuvaring, a single Annovera ring can be re-used for 13 consecutive cycles and does not require refrigeration, offering patients increased convenience with less frequent visits to the pharmacy or clinic for refills.

Annovera is a soft, flexible, silicone ring with an outer diameter of 56 mm and cross-sectional diameter of 8.4 mm that contains EE 17.4 mg and SA 103 mg with an average release rate of EE 13 mcg/day and SA 150 mcg/day [3]. The ring is placed in the vagina for 21 days continuously and removed for 7 days each cycle over one year. As with other combined hormonal contraceptive methods, the primary mechanism of action of Annovera is ovulation suppression.

Two-phase 3 studies conducted in the USA and seven international sites evaluated the efficacy, safety, and side effects of the EE/SA CVS [5•, 6•]. In these studies, investigators enrolled 2278 participants aged 18–40 years with regular cycles who had no contraindications to combined hormonal contraceptive use or anatomic abnormalities that would preclude vaginal ring use. During active use, they were instructed not to remove the CVS for more than 2 h. Over half (57.5%) completed 13 cycles; the most common reasons for early discontinuation were adverse events (AEs) related to treatment (12.3%) and loss to follow-up (9.4%).

The Pearl Index, a common technique for estimating contraceptive efficacy, is the number of contraceptive failures per 100 person-years of exposure. In participants 35 years and younger, there were 40 pregnancies in 17,427 cycles contributed by 2111 participants. The resulting overall Pearl Index of 2.98 (95% CI 2.13–4.06) for Annovera is comparable with other methods of combined hormonal contraception [5•]. The cumulative efficacy rate was 97.5% (95% CI: 96.5–98.1%), and there was no significant change in pregnancy risk across cycles, which supports efficacy over 1 year of use. Importantly, users who reported removing the CVS for longer than 2 h during active use experienced significantly higher failure rates compared with those who did not (PI 5.89 (95% CI: 3.46–9.27) versus 2.10 (95% CI: 1.37–3.06), respectively) [5•]; this finding is likely due to escape ovulation with improper use. In the pooled safety studies, 69% of participants experienced a treatment-related AE, most commonly headache (26%), nausea (18%), and vaginal discharge (10%). Four events of venous thromboembolism (VTE) occurred [6•]. Notably, only 9% of the study population had a baseline body mass index (BMI) > 29 kg/m^2^ because enrollment of this group was halted following two VTE events in obese participants. Overall, participants experienced predictable bleeding patterns with Annovera. While 13–22% of participants reported unscheduled bleeding or spotting in any cycle, less than 2% discontinued early due to bleeding complaints [7•].

In short, Annovera is an effective, long-lasting, user-controlled option that may improve contraceptive access for patients in low-resource settings or those with barriers to retrieving monthly prescriptions. Due to restricted enrollment of individuals with BMI > 29.0 kg/m^2^, the safety and efficacy of Annovera have not been adequately evaluated in this population. Because the FDA’s recommendation to include obese participants in studies did not occur until 2007 [8], most combined hormonal contraceptives were historically approved without study data that included obese people. However, given the increased VTE risk with obesity at baseline, clinicians may consider whether Annovera is an appropriate contraceptive method if there are other acceptable alternatives. To decrease risk of pregnancy with Annovera use, clinicians should counsel patients not to remove the ring for more than two hours during active use. Lastly, while other combined hormonal contraceptive methods can be used continuously without a hormone-free interval, no data currently exist to guide patients on the risks and side effects of continuous Annovera use.

## Phexxi: a Contraceptive Vaginal Gel

Although the prescription vaginal gel Phexxi® (Evofem Bio-sciences, Inc, San Diego, CA) was recently FDA-approved as a contraceptive method in May 2020, this product has been approved since 2004 as Amphora (previously known as ACIDFORM), a personal lubricant. Phexxi vaginal gel is a non-hormonal, user-controlled option for those who prefer on-demand contraception. This product joins the group of chemical barriers, which can be used alone or in conjunction with other contraceptive methods.

Phexxi is packaged as a pre-filled single-dose applicator of 5 g of gel to be administered intravaginally up to 1 h prior to each episode of vaginal intercourse [9]. The gel is a vaginal pH regulator that contains lactic acid, citric acid, and potassium bitartrate; these active ingredients maintain the naturally acidic vaginal environment to reduce sperm motility and potentially enhance the vagina’s anti-microbial defenses [10]. The pH of this gel is approximately 3.55 and has improved acid-buffering capacity compared with other vaginal gels meant for acid-buffering in the presence of alkaline semen [11]. Furthermore, Phexxi is characterized by high bioadhesive and viscosity-retaining properties in comparison to other commercial gels, which contributes to decreased product leakage from the vagina [11].

The contraceptive efficacy of Phexxi was evaluated in a multicenter, single-arm, open-label, phase 3 study over seven cycles [12•]. In this study, investigators at 112 US sites enrolled 1384 participants who were 18–35 years old, at risk of pregnancy, and had normal 21–35-day cyclic menses to use this product with at least three acts of heterosexual vaginal intercourse in each cycle. Almost half of the enrolled participants completed the study (46.7%); the study drug was used a median of 22 times per participant and median of 3.6 times per participant per cycle.

In the primary outcome of seven-cycle cumulative pregnancy rate, investigators included participants who had a 21–35-day cycle, did not use any other contraceptive, and recorded at least one act of vaginal intercourse during the cycle. Among 1114 participants with 24,289 acts of intercourse in qualifying cycles, there were 101 on-treatment pregnancies, resulting in a cumulative pregnancy rate of 13.7% (95% CI: 10.0–17.5%). Safety assessments in 1330 participants demonstrated that the most frequently reported AE was vulvovaginal burning sensation (20.0%). Other vulvovaginal complaints included pruritus (11.2%), pain (3.8%), or genitourinary tract infections, such as urinary tract infection (5.7%), mycotic infection (2.9%), and bacterial vaginosis (2.8%). Overall, serious AEs were uncommon (17, 1.3%), and only two serious AEs led to early study discontinuation. Participants were also satisfied with this product, with over 80% reporting being “very satisfied” or “satisfied” during treatment.

In addition to preventing pregnancy, Phexxi may also have microbicidal benefits, which differentiates it from other chemical barrier products. Other FDA-approved vaginal contraceptives contain nonoxynol-9 (N-9), a surfactant that disrupts cell membranes. These vaginal spermicides are available without prescription and are found in a variety of delivery systems, including gels, creams, foams, suppositories, films, and sponges. The contraceptive efficacy of N-9 spermicidal products ranges from 10.0 to 22.2% with typical use over a 6-month period, with lower dose products having higher failure rates [13, 14]. Vaginal contraceptive products containing N-9 had previously been thought to also exhibit microbicidal activity; however, studies have demonstrated that N-9 does not reduce the risk of gonorrhea, chlamydia, trichomoniasis, bacterial vaginosis, or candidiasis compared with placebo and could potentially increase HIV acquisition with frequent applications [15, 16]. In contrast to N-9-based vaginal contraceptive products, Phexxi has the potential to have both microbicidal and contraceptive properties in one product due to its mechanism of maintaining the acidic environment of the vagina to enhance its natural antimicrobial defenses. In a double-blind, placebo-controlled, multicenter phase 2B/3 study in US women who had been diagnosed with chlamydia or gonorrhea, participants who received Phexxi had a relative risk reduction of 50% and 80% of chlamydia and gonorrhea infections, respectively [17•]. A phase 3 trial (NCT04553068) is currently in process for the evaluation of Phexxi in preventing urogenital chlamydia and gonorrhea infections compared with placebo.

In summary, Phexxi is a user-controlled, non-hormonal, on-demand contraceptive method that represents a novel addition to the contraceptive market with its additional uses as a personal lubricant and as a potential microbicide for urogenital infection prevention. However, Phexxi requires prescription, which may limit access in context of other available non-prescription vaginal contraceptive products.

## Slynd: a Drospirenone-Only Pill

Progestin-only pills (POPs) are one option for patients who have contraindications to estrogen use; these include patients with hypertension, cardiovascular disease risk factors, migraines with aura, and risk factors for VTE, including those in the early postpartum period [18]. Although POPs have comparable efficacy and fewer contraindications than combined oral contraceptives (COCs), only 0.4% of all US reproductive-aged women used POPs between 2006 and 2010 [19]. Several well-known disadvantages of POPs likely contribute to its low prevalence of use. Prior to FDA approval of Slynd® (Exeltis USA, Inc, Florham Park, NJ) in 2019, the only available POP in the USA contained norethindrone 0.35 mg, a progestin dose that is lower than what is included in COCs. As opposed to consistent ovulation suppression obtained with combined hormonal contraception, the norethindrone pill suppresses ovulation in approximately half of users [20, 21]. Instead, it primarily prevents pregnancy by thickening the cervical mucus to block sperm migration along with affecting the endometrium or tubal motility. Accordingly, the 3-h missed pill window for norethindrone pills requires strict adherence to maintain efficacy [20]. Available outside of the USA, desogestrel pills have an increased missed pill window of 12 h [22]. However, neither product compares to the flexibility of the 24-h missed pill window for COCs. Furthermore, norethindrone POPs are taken continuously without a hormone-free interval resulting in high rates of method discontinuation due to irregular bleeding [23].

Slynd, a new progestin-only contraceptive pill, attempts to address these drawbacks. Slynd contains drospirenone 4 mg, a higher dose than what is found in COCs. Drospirenone is an analog of spironolactone with antimineralcorticoid activity and a long half-life of 25–30 h. Due to its prolonged half-life, the drospirenone-only pill has the potential to improve irregular bleeding and increase dosing flexibility by increasing the missed pill window. Slynd comes in a 28-day pack with 24 days of hormonal pills and 4 days of inert pills; the hormone-free period is intended to produce a scheduled withdrawal bleed [24•, 25]. The primary mechanism of Slynd is ovulation suppression [26].

The contraceptive efficacy, safety, and tolerability of oral drospirenone 4 mg over 13 cycles were evaluated in an open-label phase 3 trial at 41 US sites [24•]. Investigators included 1006 healthy, sexually active participants who were 15 years or older with regular menstrual cycles. Lactating women who were at least 6 weeks postpartum were included for safety evaluations. Sixty-five percent of participants completed the study; the most common reasons for early discontinuation were loss to follow-up (26.7%) and withdrawal of consent (10.4%). Seventeen pregnancies occurred in 5547 cycles contributed by 953 participants, resulting in the Pearl index of 4.0 (95% CI: 2.3–6.4). Approximately one-third of study participants were obese (BMI ≥ 30.0 kg/m^2^), and the Pearl Index was not affected by BMI.

In terms of safety assessments, 11.2% discontinued early from the study due to AEs. Given the antimineralcorticoid activity of drospirenone, participants were monitored for hyperkalemia; five (0.5%) participants had asymptomatic hyperkalemia. No VTE events occurred during study participation. With continued Slynd use, the proportion of participants reporting amenorrhea increased and unscheduled bleeding decreased, although approximately 40% of participants still had unscheduled bleeding in cycles 11–13. Regardless, few (1.9%) discontinued early due to bleeding complaints. Furthermore, about 85% of users reported they were satisfied with the product [24•].

Overall, Slynd improves upon the major challenges of POP use. This pill provides more flexibility with delayed or missed pills for users while maintaining efficacy comparable to other contraceptive pills. The cyclic regimen may not result in regular bleeding patterns for all users, but there is evidence that the bleeding profile with Slynd may be more favorable than with previously available POPs. Slynd is an attractive option for patients who need, or wish, to avoid estrogen-containing contraceptives and desire to use oral contraceptive pills for pregnancy prevention.

## Twirla: a Combination Contraceptive Patch

Transdermal delivery of contraceptive hormones offers users multiple benefits compared with pill administration, including more stable hormone levels, decreased loss of drug bioavailability with avoidance of hepatic first-pass metabolism, and an alternative to daily dosing with the potential to improve compliance. The first contraceptive patch, Ortho Evra® (Janssen Pharmaceuticals, Inc, Titusville, NJ), was approved in the USA in 2001 and contained EE 0.75 mg and norelgestromin (NGMN) 6 mg, releasing a daily dose of EE 35 mcg and NGMN 150 mcg [27]. This patch is no longer available after introduction of the generic version, Xulane® (Mylan Pharmaceuticals, Inc, Canonsburg, PA), in 2014.

The EE/NGMN patch carries safety and efficacy concerns. When comparing the pharmacokinetic parameters for the EE/NGMN patch with an oral contraceptive pill containing EE 35 mcg and norgestimate 250 mcg, the area under the curve was approximately 55% higher in patch compared with pill users, leading to concerns over an increased risk of venous thromboembolism (VTE) with the additional EE exposure [27]. Despite the pharmacokinetic finding, epidemiologic data are conflicting as to whether VTE risk with patch use is increased compared with combined oral pills [28]. With respect to efficacy, one-third of the on-treatment pregnancies occurred in participants who were ≥ 90 kg, suggesting a decreased efficacy with increasing body weight [29].

Twirla® (Agile Therapeutics, Inc, Princeton, NJ) was developed to address the need for a lower-dose product that would maintain the same benefits of a transdermal system. After FDA approval in February 2020, Twirla became the second available contraceptive patch on market. This combination contraceptive patch contains EE 2.3 mg and LNG 2.6 mg releases a daily dose of EE 30 mcg and LNG 120 mcg [30]. The total patch area measures 28 cm^2^ with the center active adhesive laminate measuring 15 cm^2^. A new Twirla patch is applied weekly on the abdomen, buttock, or upper torso for three consecutive weeks followed by a 7-day patch-free interval. Similar to other combined hormonal contraceptives, this product has the same primary mechanism of action of ovulation suppression. In contrast to the increased EE exposure with the EE/NGMN patch, the maximum concentration and steady-state concentration of Twirla were 60% and 18% lower, respectively, compared with the EE/norgestimate oral contraceptive pill [31].

The contraceptive efficacy, safety, and tolerability of Twirla were evaluated in a single-arm, open-label, phase 3 study in 102 US sites [32•]. Investigators enrolled 2032 participants who were 18 years and older, had regular 21 to 38 day cycles, were at risk of pregnancy, and did not have any contraindications to combined hormonal contraceptive use based on the 2016 US Medical Eligibility Criteria for Contraceptive Use. Participants were instructed to use Twirla as their sole contraceptive method over 13 cycles, with each cycle consisting of three consecutive weekly patches followed by one patch-free week. About half of the participants (48.7%) completed the study, with most participants discontinuing early due to participant desire (15%), loss to follow-up (11%), and AE (11%). The BMI distribution of participants was 39% normal weight, 25% overweight, and 35% obese.

In the primary efficacy analysis, there were 68 pregnancies in 15,165 cycles contributed by 1736 participants who were 35 years and younger, resulting in an overall Pearl Index of 5.8 (95% CI 4.5–7.2) pregnancies per 100 woman-years. Notably, efficacy differed by BMI, with Pearl indices of 3.5 (95% CI 1.8–5.2), 5.7 (95% CI 3.0–8.4), and 8.6 (5.8–11.5) among normal, overweight, and obese participants, respectively. As with other combined hormonal contraceptive methods, scheduled bleeding is expected with Twirla. The proportion of participants with amenorrhea ranged from 6.3 to 11.9% while the proportion of participants reporting one or more days of unscheduled bleeding and spotting decreased from 60% in cycle 1 to 42% in cycle 13. Among 2031 participants in the safety analysis, 27% experienced a study-related AE; the most frequently reported hormone-related AEs were nausea (4.1%) and headache (3.6%). Five participants, all obese, experienced six venous thromboembolism events; one event was not considered to be related to the study drug. Lastly, Twirla had high wearability with only 5% of all patches completely detaching during the study period. Participants reported a decrease in patch-associated moderate or severe irritation and itching over time.

Overall, Twirla offers users an additional option for a reversible, user-controlled, combined hormonal contraceptive method that does not require daily dosing. However, the potential advantages of an increased safety profile with the lower EE exposure seen with Twirla compared to its predecessor were not realized as the risks of VTE are likely in the same range as other combined hormonal contraceptive methods at 3 to 12 per 10,000 women [30]. The occurrence of VTE coupled with increased failure rates among obese participants limits Twirla prescription to people with BMI ≤ 30 kg/m^2^. Lastly, while Twirla use is not contraindicated in overweight individuals, clinicians should also be aware of and counsel patients regarding the slightly increased failure rates in this group as well.

## Conclusions

In this review, we described the efficacy, side effects, risks, and benefits of four newer products on the US contraceptive market, specifically Annovera vaginal ring, Phexxi vaginal gel, Slynd drospirenone-only pill, and Twirla transdermal patch, which are summarized in Table 1. While the overall prevalence of short-acting hormonal and coitally-dependent contraceptive method use appears to be decreasing [33], the addition of these products to the contraceptive method mix could potentially fill any gaps between patient needs and existing products.

Despite these recent innovations, there is still room for further contraceptive development to expand options within the US and global markets. Male contraception remains an area with significant potential to change the contraceptive landscape as male condoms and vasectomy are the only currently available methods. Another avenue of importance is technologies that address multiple needs in a single product, such as contraception and infection prevention. As obesity rates continue to increase, developing methods that are safe and effective in obese individuals is an important priority. Lastly, a gap exists for short-acting, user-controlled, non-hormonal contraceptive methods that are not coitally dependent. No single contraceptive product is right for everyone; expanding options provides patients with increased flexibility when choosing methods that will work for their needs and preferences.

## Figures and Tables

**Table 1:** Product summary, efficacy, safety, benefits, and additional considerations of Annovera, Phexxi, Slynd, and Twirla

	Product summary	Efficacy	Safety/Adverse Events	Benefits	Additional considerations
Annovera	Contraceptive vaginal system releasing ethinyl estradiol 13 mcg/day and segesterone acetate 150 mcg/day	Pearl Index 2.98 (95% CI 2.13–4.06) Cumulative efficacy rate of 97.5% (95% CI: 96.5–98.1%)	Most common adverse events were headache, nausea, and vaginal discharge Limited evidence for BMI > 29.0 kg/m^2^ given exclusion of participants after two venous thromboembolism events occurred	1 ring can be used for 13 cycles	Cannot remove ring for more than 2 h during active use No evidence for continuous ring use without cyclic hormone-free interval
Phexxi	5 g of vaginal gel containing lactic acid, citric acid, and potassium bitartrate, pH regulator	Cumulative pregnancy rate of 13.7% (95% CI: 10.0–17.5%)	Most common adverse events were vulvovaginal burning sensation (20.0%), pruritus (11.2%), and urinary tract infections (5.7%)	On-demand use with intercourse, additional functions of personal lubricant	Currently being evaluated as a microbicide
Slynd	Drospirenone 4 mg pill	Pearl Index 4.0 (95% CI: 2.3–6.4)	Few cases of hyperkalemia (0.5%) Safe for those with contraindications to estrogen use	24-h missed pill window	Unscheduled bleeding in approximately 50% of users
Twirla	Contraceptive patch releasing ethinyl estradiol 30 mcg/day and levonorgestrel 120 mcg/day	Pearl Index 5.8 (95% CI: 4.5–7.2)	Most common adverse events were nausea (4.1%) and headache (3.6%) Five venous thromboembolism events occurred in obese participants	Weekly dosing	Lower efficacy in overweight compared with normal weight individuals
